# Genomic Differentiation and Demographic Histories of Atlantic and Indo-Pacific Yellowfin Tuna (*Thunnus albacares*) Populations

**DOI:** 10.1093/gbe/evx067

**Published:** 2017-04-01

**Authors:** Julia M.I. Barth, Malte Damerau, Michael Matschiner, Sissel Jentoft, Reinhold Hanel

**Affiliations:** 1 Centre for Ecological and Evolutionary Synthesis (CEES), Department of Biosciences, University of Oslo, Oslo, Norway; 2 Institute of Fisheries Ecology, Johann Heinrich von Thünen Institute, Federal Research Institute for Rural Areas, Forestry and Fisheries, Hamburg, Germany; 3 Centre for Coastal Research, Department of Natural Sciences, University of Agder, Kristiansand, Norway

**Keywords:** population genomics, whole-genome sequencing, demography, conservation, tuna, fisheries management

## Abstract

Recent developments in the field of genomics have provided new and powerful insights into population structure and dynamics that are essential for the conservation of biological diversity. As a commercially highly valuable species, the yellowfin tuna (*Thunnus albacares*) is intensely exploited throughout its distribution in tropical oceans around the world, and is currently classified as near threatened. However, conservation efforts for this species have so far been hampered by limited knowledge of its population structure, due to incongruent results of previous investigations. Here, we use whole-genome sequencing in concert with a draft genome assembly to decipher the global population structure of the yellowfin tuna, and to investigate its demographic history. We detect significant differentiation of Atlantic and Indo-Pacific yellowfin tuna populations as well as the possibility of a third diverged yellowfin tuna group in the Arabian Sea. We further observe evidence for past population expansion as well as asymmetric gene flow from the Indo-Pacific to the Atlantic.

## Introduction

High-throughput sequencing technology is a valuable tool for the conservation and management of species and populations (Ouborg et al. 2010) enabling the discovery of fine-scale genetic variation and thus the deduction of population divergences with drastically increased accuracy (Hemmer-Hansen et al. 2014). Even for marine species with high gene flow and low population genetic structuring, it is now possible to precisely identify biologically relevant populations by their signatures of local adaptation, and to decipher the demographic imprints caused by population size changes or dispersal (Nielsen et al. 2009; Allendorf et al. 2010). For sustainable management of marine fish, identifying management units (stocks) concordant with biological population units defined by their adaptive potential to environmental variables is of prime importance. Any mismatch will exacerbate the increasing trend of unsustainably harvested fish stocks that suffer severe population declines or even collapse (Mullon et al. 2005; Reiss et al. 2009). This is particularly important for intensively harvested species of high economic value, for which population size declines may be exceptionally steep (Juan-Jordá et al. 2011). However, overall population differentiation of pelagic fishes like tunas, characterized by wide geographical distributions, large population sizes, and high dispersal capabilities, is usually low due to high levels of gene flow, thus limiting the applicability of classical genetic markers such as allozymes, mitochondrial DNA, or microsatellites (Hauser and Ward 1998). Differentiation between populations connected by gene flow is often limited to genomic regions underlying traits involved in local adaptation (Funk et al. 2012) that are easily missed when only few markers are employed. By identifying vast numbers of single nucleotide polymorphisms (SNPs) distributed throughout the genome, high-throughput sequencing approaches can capture this adaptive variation and thus help to resolve population structure in the presence of gene flow.

The genus *Thunnus* encompasses eight economically important large tuna species, with the yellowfin tuna (*Thunnus albacares*, Bonnaterre 1788) representing the main source of fishery catch in this group, exceeding 1.2 million tons in 2013 (Collette and Nauen 1983; ISSF 2015). Currently, four different Tuna Regional Fisheries Management Organizations globally manage four yellowfin tuna units, namely the Atlantic, Indian, Eastern Pacific, and Western Central Pacific stocks, which are fully exploited or may even be overexploited (Majkowski 2007). The International Union for Conservation of Nature (IUCN) categorizes the yellowfin tuna as near threatened with a decreasing population trend (Collette et al. 2011). High fishing pressure, accompanied by population decline, highlights the need for a thorough understanding of the ecology of yellowfin tuna and for tools capable of tracing illegally caught fish in order to develop more sustainable management.

The population structure of yellowfin tuna has already been investigated with a variety of approaches, including morphometric and meristic characters (Royce 1964; Schaefer 1991), otolith microchemistry (Gunn and Ward 1994), fisheries statistics (e.g., Suzuki et al. 1978), tagging data (Itano and Williams 1992; Ortiz 2001; Fonteneau and Hallier 2015), and genetics (e.g., Ward et al. 1997; Ely et al. 2005). While the nonmolecular studies support population structuring of yellowfin tuna within the Atlantic, Indian, and Pacific Oceans, the results of the genetic analyses are less congruent and are largely dependent on the choice of markers. For instance, a study on the global population genetic variation in allozymes and mitochondrial restriction fragment length polymorphisms (RFLP) revealed that separate yellowfin tuna populations exist in the Atlantic, Indian, and Pacific Oceans (Ward et al. 1997), while another study based on mitochondrial D-loop sequences and RFLPs found no significant evidence for distinct populations on a global scale (Ely et al. 2005). Within oceans, the results of genetic studies are even more diverse, ranging from the identification of distinct populations within a relatively confined area along the coast of India and Sri Lanka based on D-loop sequences (Kunal et al. 2013) to the observation of homogeneity across large spatial scales in microsatellites and D-loop sequences for pan-Pacific samples (Nomura et al. 2014). While the significant genetic differentiation observed in some studies points towards the existence of subpopulations between and within oceans, the lack of conclusive results emphasizes the necessity for a revision of the yellowfin population structure using more powerful genomic tools. Indeed, preliminary analyses derived from reduced-representation genome sequencing indicated differentiation among Atlantic, Indian, and Pacific populations (Pecoraro et al. 2015). Within the Pacific Ocean, selected SNPs putatively under positive selection were used to distinguish unambiguously between western, central, and eastern populations, while no genetic structure was detected using neutral loci (Grewe et al. 2015). These results provide further support for the existence of important genetic diversity in the yellowfin tuna, which demands an in-depth assessment for the conservation and management of this commercially and otherwise valuable species.

Inference of demographic history can help to understand responses to past environmental changes, making them relevant for management strategies. Despite considerable fisheries-induced population size declines within the last decades (Juan-Jordá et al. 2011), high catch rates and the seemingly low genetic differentiation across wide geographic scales indicate large effective population sizes of yellowfin tuna. In practice, relative abundances of yellowfin tuna within management areas are usually estimated from annual catch rates, but genetic estimates of the effective population size (*N*_e_) offer insights into the global long-term demography. Ely et al. (2005) calculated the *N*_e_ of females with mismatch distributions of D-loop sequences to be about ten million individuals, with a historical population expansion around 500 ka. This time line implies little influence of past glacial cycles on this oceanic species adapted to tropical and subtropical regimes, in contrast to neritic species that underwent considerable population declines during glacial maxima (Hewitt 2004).

The identification of biological population units for the management of yellowfin tuna is a timely but challenging task for which low discriminative power has been a limiting factor in previous studies. Therefore, we apply a whole-genome resequencing approach here in combination with a yellowfin tuna draft genome assembly to delineate major populations within the species’ circumglobal range based on a large number of unbiased SNPs distributed across the genome. Demographic comparisons of these populations revealed past population expansion as well as asymmetric gene flow from the Indo-Pacific into the Atlantic, potentially driven by warm-water ocean currents.

## Materials and Methods

### Sample Collection

**Fig. 1.—:**
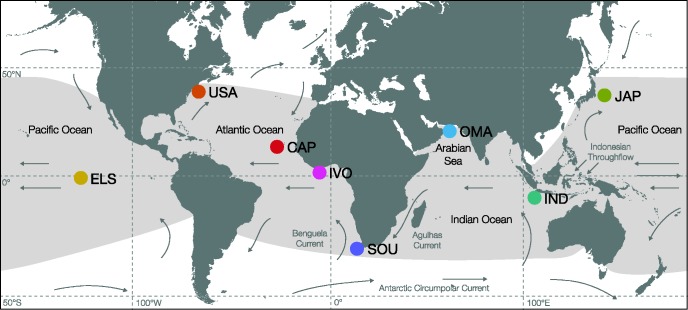
Circumtropical distribution of yellowfin tuna (light-gray shaded area) and sampling sites for this study (colored circles): USA (U.S.A.), CAP (Republic of Cabo Verde), IVO (Ivory Coast), SOU (South Africa), OMA (Oman), IND (Indonesia), JAP (Japan), and ELS (El Salvador). Arrows indicate major ocean surface currents.

Specimens were sampled at 8 localities, covering most of the global distribution of the yellowfin tuna: Rhode Island, U.S.A. (USA, *N* = 3); Mindelo, Republic of Cabo Verde (CAP, *N* = 5); Abidjan, Ivory Coast (IVO, *N* = 6); Cape Town, South Africa (SOU, *N* = 7); Barka, Oman (OMA, *N* = 5); Denpasar, Indonesia (IND, *N* = 3); Sagami Bay and Okinawa, Japan (JAP, *N* = 11); central-eastern Pacific, El Salvador (ELS, *N* = 2; fig. 1 and supplementary table S1, Supplementary Material online).

### DNA Extraction and Sequencing

Genomic DNA was extracted from muscle tissue or fin clips using the E.Z.N.A Tissue DNA kit (Omega Bio-Tek, Norcross, GA, USA) according to the manufacturer’s protocol. DNA quality and quantity were assessed with the Qubit dsDNA BR assay (Life Technologies, Carlsbad, CA, USA), through visual inspection of agarose gels, and by quantitation using a 2100 Bioanalyzer (Agilent Technologies, Santa Clara, CA, USA). Illumina sequencing libraries (TruSeq DNA PCR-Free Library Preparation Kit, Illumina, San Diego, CA, USA) were prepared at the Norwegian Sequencing Centre (NSC, www.sequencing.uio.no; last accessed April 7, 2017) according to the center’s protocols. In brief: DNA was normalized to 20 ng/µl and fragmented using a focused-ultrasonicator (E220, Covaris, Woburn, MA, USA) for a 350 base-pair (bp) insert size (duty factor: 10%, peak incident power: 175 W, cycles per burst: 200, duration: 50 s, mode: frequency sweeping, temperature: 5.5–6 °C). The resulting fragment sizes were checked on the 2100 Bioanalyzer. Fragments were cleaned and adaptors were ligated following Illumina’s recommendations. The final libraries were eluted in 22.5 µl Illumina resuspension solution and stored at −20 °C until clustering and 125 bp paired-end sequencing on an Illumina HiSeq 2500 (Illumina, San Diego, CA, USA). The final dataset results from three independent sequencing runs that were conducted to increase coverage and avoid technical bias. For all runs, demultiplexed sequences were received from the NSC and sequence quality as well as key information such as GC content, overrepresentation of adaptors and average length were checked with the software fastqc v0.11.2 (http://www.bioinformatics.babraham.ac.uk/projects/fastqc; last accessed April 7, 2017).

### Mapping and Variant Calling

All reads were mapped using the algorithm BWA-MEM in bwa v0.7.8 (Li and Durbin 2009) against a de-novo assembled yellowfin tuna draft genome sequence (estimated genome size: 836 Mb, coverage: 18.6×, scaffold N50: 46,871 kb, CEGMA scores: complete 209, partial 236; for details see Malmstrøm et al. [2016]). The draft genome assembly was indexed with bwa, samtools v1.1 (Li and Durbin 2009; Li 2011), and picard-tools v1.107 (http://broadinstitute.github.io/picard; last accessed April 7, 2017). Mapped files were converted to BAM format, sorted, and indexed using samtools. Duplicates were marked using picard-tools, and indels were realigned using gatk v3.2.2 (McKenna et al. 2010). In cases with more than one library per individual, the mapped reads were merged using picard-tools followed by samtools’ sorting, picard-tools’ deduplication, and gatk’s indel realignment. Mean and median read coverages calculated with bedtools v2.25.0 (Quinlan and Hall 2010) were 9.24 ± 4.21× and 7.81 ± 3.38×, respectively (averaged across samples, ± standard deviation). None of the samples had a mean or median coverage below 5×. One sample from each of the Atlantic and Pacific Oceans was sequenced at higher coverage (means: 21.67× and 22.24×, medians: 20× and 21×, respectively).

Variants were called using freebayes v0.9.14 (Garrison and Marth 2012) and gatk v3.3.2 (DePristo et al. 2011). gatk detects variants in a two-step process, first within single samples, followed by a joint genotyping analysis, whereas freebayes does not include a per-individual analysis step. The resulting variant calls (freebayes: 36,236,249 variants, gatk: 38,033,064 variants) were further filtered to include only biallelic SNPs, and to remove SNPs within 10 bp of an indel. The gatk file was hard filtered according to gatk’s recommendations: FS > 60.0, MQRankSum < −12.5, ReadPosRankSum < −8.0, QD < 2.0, MQ < 40.0. The intersection of the two different filtered SNP sets (freebayes: 24,693,365 SNPs, gatk: 22,479,358 SNPs) was identified using the isec command in bcftools’ v1.2 (Li 2011), and all other SNPs were excluded from further analysis. In the resulting SNP set, all sites with a genotype quality score < 20, and read depth (DP) < 3, or DP > 100 were replaced with missing data for the respective individual. In addition, the scaffold containing the mitochondrial genome was excluded. The remaining 17,845,442 SNPs were further filtered using a special build of the software plink v1.90 beta (https://www.cog-genomics.org/plink2; last accessed April 7, 2017; Purcell et al. 2007), which allows larger “chromosome” numbers with the flag “- -aec” (plink_high_contig, available from the software authors): We excluded SNPs displaying a minor-allele count < 2 across all populations and SNPs deviating from Hardy–Weinberg equilibrium with a *P* value < 0.0001, but only when the deviation was due to significant heterozygote excess (*P* value < 0.0001). Additional filtering on linkage disequilibrium (LD, squared correlation coefficient (*r^2^*) > 0.8), minor-allele frequency (MAF, threshold between 1 and 3%), and missing data per site (allowing a maximum of 10, 20, or 50%) was performed using plink, depending on the downstream analysis (see respective sections below). The cut-off for LD was determined by estimating linkage decay: *r^2^* was calculated between pairs of SNPs with maximally 20% missing data and MAF > 1% for all individuals using plink. We used the flags “- -ld-window 10000”, “- -ld-window-kb 10000”, and “- -ld-window-r2 0” to force pairwise comparisons between all markers per scaffold, which resulted in 3,808,567 *r^2^* values. The *r^2^* values were assigned to 1-kb bins according to their physical distance and the mean *r^2^* per bin plotted using r v3.1.0 (R Core Team, 2015; supplementary fig. S1, Supplementary Material online).

Mitochondrial reads were mapped and indexed as described above against a fully assembled yellowfin tuna mitochondrial genome (Guo et al. 2014). Mitochondrial SNPs were called using mpileup in samtools v1.3, applying a minimum mapping quality (“-q 20”) and a minimum base quality (“-Q 30”) filter before extracting the consensus sequence using the “-c” flag in the call command in bcftools v1.3. The resulting variant call consensus sequence was transformed into fastq format using vcfutils (vcf2fq) in samtools only when the minimum depth was above 6×. The fastq format was translated into fasta format using seqtk v1.0-r75 (https://github.com/lh3/seqtk; last accessed April 7, 2017).

### Species Verification

We complemented morphological species identification of our specimens by comparison of the entire mitochondrial genomes with 26 mitochondrial genomes of all eight species of the genus *Thunnus* and one outgroup (*Katsuwonus pelamis*), retrieved from GenBank (Benson et al. 2013). All sequences were jointly aligned using the “- -auto” option in Mafft v7.158b (Katoh et al. 2002) and the alignment was visually checked using Aliview v1.16 (Larsson 2014). A maximum likelihood tree search with ten individual runs was performed under the GTRCAT substitution model using raxml v8.0.26 (Stamatakis 2014), and node support was assessed with up to 1,000 bootstrap replicates (option “autoMRE”; supplementary fig. S2, Supplementary Material online).

### Genetic Differentiation

**Fig. 2.—:**
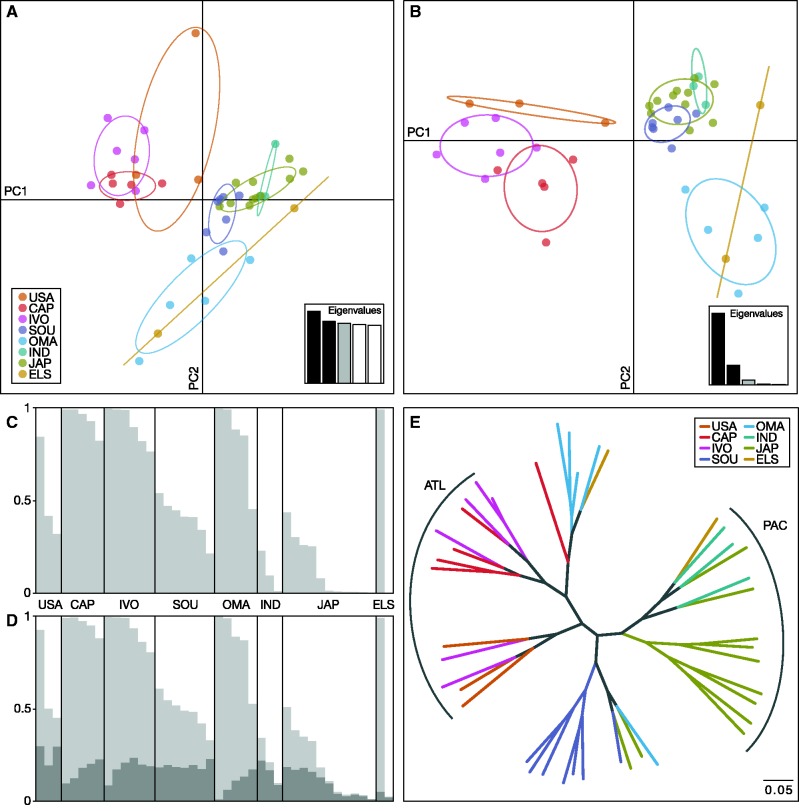
Genetic differentiation of yellowfin tuna. (*a*) Principal component analysis (PCA) showing a separation of Atlantic (USA, CAP, and IVO) and Indo-Pacific samples (SOU, IND, and JAP) on the first principal component axis (PC1) and additional differentiation of the Arabian Sea samples (OMA) on PC2. (*b*) Discriminant analysis of principal components (DAPC) describing the variation between the sampling sites. In (*a*) and (*b*), inertia ellipses summarize the variation per sampling site (using adegenet’s default “cellipse” value of 1.5, these correspond to 67% confidence intervals) and eigenvalues for the first five PCs are displayed in bar plot insets. (*c*, *d*) Individual admixture bar plots. structure*q* values (vertical axis) are shaded according to cluster membership, black lines separate sampling locations. Within sampling sites, individuals are sorted according to assignment proportions. Number of tested clusters: (*c*) *K* = 2, (*d*) *K* = 3. (*e*) Maximum-likelihood phylogenetic clustering of individuals. Groups including only Atlantic individuals are marked with “ATL”, groups including only Indo-Pacific individuals with “PAC.” The scale bar indicates the number of substitutions.

Pairwise fixation indices (*F*_ST_) were estimated according to Weir and Cockerham (1984) by applying the “- -weir-fst-pop” flag in vcftools v0.1.14 (Danecek et al. 2011), and according to Nei (1987) using the package hierfstat v0.04-22 (Goudet 2005) in r, based on SNPs with MAF > 1% and at most 10% missing genotypes (supplementary table S2, Supplementary Material online). Bootstrapping was performed to obtain confidence intervals (95%) and *P* values for Weir & Cockerham’s *F*_ST_ estimates using the r package StAMPP (Pembleton et al. 2013), and *P* values were adjusted for multiple testing by applying sequential Bonferroni correction (Rice 1988). Similar *F*_ST_ estimates were obtained with other filtering thresholds for MAF and missing data (supplementary table S3, Supplementary Material online). Heterozygosity and the inbreeding coefficient (*F*_IS_) were calculated using the package hierfstat. Genetic differentiation between sampling localities was assessed with principal component analysis (PCA) and discriminant analysis of principal components (DAPC) of a SNP set with MAF > 1% and at most 10% missing genotypes using the package adegenet v1.4-1 (Jombart 2008; Jombart et al. 2010) in r. This analysis was performed separately with data from all samples, from Atlantic individuals only, and with Indo-Pacific individuals only. To prevent overfitting of the DAPC, the number of retained principal components (PCs) was chosen according to the optimal α-score. We retained five of 40 PCs for the analysis including all sampling sites (see fig. 2*b*), four of 13 for the comparison among Atlantic sampling sites, and five of 14 for the comparison among Indo-Pacific sites (supplementary fig. S3*a* and S3*b*, Supplementary Material online). Significance of cluster separation was tested using a one-way analysis of variance (ANOVA) in r. Individual ancestry and the most appropriate number of genetic clusters (*K*) was assessed using the Bayesian clustering method implemented in structure v2.3.4 (Pritchard et al. 2000) under the admixture model with correlated allele frequencies for closely related or highly migratory species (Falush et al. 2003). Default values were applied for the correlated allele frequency model prior (mean: 0.01, standard deviation: 0.05). Five replicates were performed, each testing for one to five clusters (*K *= 1 to *K *= 5) using SNPs in linkage equilibrium (*r^2^* ≤ 0.8) with MAF > 3% and at most 20% missing genotypes. The Markov-chain Monte Carlo (MCMC) was run for 800,000 generations of which the first 300,000 generations were discarded as burn-in. To account for putative biases in the inference of the true number of *K* by uneven sample sizes (Puechmaille 2016), cluster membership was also investigated with an even sampling scheme of 5 individuals each from CAP, SOU, OMA, and JAP, using the same model assumptions and parameters. raxml was used to construct the maximum-likelihood tree under the GTR model with ascertainment bias correction for the absence of invariant sites (“-m ASC_GTRCAT”; Lewis 2001). For this analysis, a larger set of SNPs with MAF > 3% and maximally 50% missing data per site was used to improve the phylogenetic accuracy by character additions irrespective of an increased amount of missing data (Jiang et al. 2014). Rate heterogeneity was disabled using the flag “-V”. Node support was assessed with 100 bootstrap replicates.

To generate a mitochondrial haplotype genealogy graph, the mitochondrial genomes were aligned using default settings in mafft and alignments were visually checked and corrected using aliview. A parsimony tree was obtained using raxml and used to visualize haplotype relationships with fitchi v1.1.4 (Matschiner 2016). To reduce graph complexity, transitions were ignored in the calculation of edge lengths by applying the “-x” flag. The parsimony tree search was repeated ten times with different random number seeds; all haplotype genealogy graphs constructed from these trees were qualitatively similar.

### Demography and Gene Flow

To detect differences in the demographic histories of populations, a pairwise sequentially Markovian coalescent (PSMC; Li and Durbin 2011) analysis was performed with the two high-coverage genomes representing the Atlantic (CAP248) and Indo-Pacific (IND569) populations. We inferred heterozygous sites following Li and Durbin (2011): on each genome separately, SNP calling was performed using samtools’ mpileup command, applying a minimum mapping quality (“-q 20”) and a minimum base quality (“-Q 30”) threshold, and the consensus sequence was extracted using the “-c” flag in the call function of bcftools. The resulting consensus sequence was transformed into fastq format using the function vcfutils (vcf2fq) in samtools only when the minimum depth was above 6× and the maximum depth did not exceed 40×. The fastq file was converted into PSMC input format using the tool fq2psmcfa by choosing a minimum quality threshold (“-q”) of 20, a “scaffold-good-size” (“-g”) of 10,000 bp, and a window size (“-s”) of 20. The optimal window size is dependent on the density of heterozygous sites and was adjusted so that the number of windows containing two or more SNPs is ∼1% of all windows. Subsequently, the population history was inferred by running PSMC for 25 iterations with the following parameters: “-N 15”, “-r 5”, and “-p 4*4 + 13*2 + 4*4 + 6.” In order to prevent overfitting, we reduced the number of free parameters to 22. Bootstrapping (100 replicates) was performed to assess uncertainty in the PSMC estimates using the splitfa script provided with the software. The trunk size was adjusted corresponding to the scaffold size and set to 1 Mb. The output of the PSMC needs to be scaled using an estimate of mutation rate per generation and a generation time in years. According to IUCN, the generation time of yellowfin tuna is 2.2–3.5 years (Collette et al. 2011). Estimates of the genome-wide yellowfin tuna mutation rate were not available and were therefore computed based on the divergence time and number of substitutions between the genome sequences of the yellowfin tuna (Malmstrøm et al. 2016) and the closely related Pacific bluefin tuna (*Thunnus orientalis*, Nakamura et al. 2013). The divergence time between these two species was recently estimated to be 1.9965 Ma based on a fossil-calibrated phylogeny of teleost genome sequences (Musilová et al., unpublished). To map the yellowfin tuna reads against the Pacific bluefin tuna genome, samtools’ mpileup function was used with the same filter sets as above, which resulted in 9.24 × 10^6^ substitutions. Assuming a generation time of 2.5 years and a genome size of 684 Mb (Pacific bluefin tuna; Nakamura et al. 2013), this translates to a mutation rate (*μ*) of 7.3 × 10^−9^ substitutions/site/generation. If instead a generation of 3.5 years is assumed, the resulting mutation rate is 1.0 × 10^−8^ substitutions/site/generation. Generally, nuclear SNPs are assumed to have mutation rates on the order of 10^−8^ to 10^−9^ substitutions/site/generation (Brumfield et al. 2003), and the human mutation rate is estimated to be 2.5 × 10^−8^ substitutions/site/generation (Nachman and Crowell 2000), which would place our estimates at the slower end of the scale. Demographic histories inferred from phylogenetically derived mutation rates may overestimate the timing of past population size changes (Ho et al. 2005), however, these rates can serve as minimum estimates with the real mutation rate possibly being larger. To account for this potential bias, we performed demographic analysis with three mutation rates (per site per generation): 7.3 × 10^−9^ (the minimum estimate obtained using a generation time of 2.5 years), 2.5 × 10^−8^ (the established human mutation rate), and 1.5 × 10^−8^ (an intermediate estimate).

To estimate demographic parameters while taking into account potentially asymmetric rates of gene flow between Atlantic and Indo-Pacific populations, we performed coalescent simulations with the software fastsimcoal2 v.2.5.2 (Excoffier et al. 2013) according to an evolutionary scenario in which an ancestral population splits into two extant populations that are connected by bi-directional gene flow. The divergence event between the two extant populations was assumed to be instantaneous, the effective population sizes of ancestral and extant populations were assumed to be constant, and the mutation rate was fixed to 7.3 × 10^−9^ per site and generation (see above). The divergence time, the three effective population sizes, and the two migration rate parameters in these simulations were optimized so that the joint site-frequency spectra (SFS) resulting from simulated sequence data were similar to the observed joint SFS. For this comparison, the minor-allele SFS of 14 Atlantic (USA, CAP, and IVO) and 16 Indo-Pacific (IND, JAP, and ELS) yellowfin tuna was calculated from all sites with a maximum of 50% of missing data per group (Atlantic and Indo-Pacific), by drawing at random 14 alleles per group. In cases where the frequency of the two allelic states was exactly 0.5, both were used for the joint SFS with a weight of 0.5 (see Roesti et al. 2015). To account for a potential bias caused by the heterogeneity of the ELS sample, all parameters were additionally calculated by only considering IND and JAP as Indo-Pacific population. Prior distributions for the six parameters were set as in Roesti et al. (2015). We carried out 80 replicate sets of simulations, each including 40 estimation loops with 100,000 simulations. Out of the 80 sets of simulations, we used the ten best-fitting sets (those with the smallest difference between the estimated and the observed likelihood) to calculate mean estimates of demographic parameters.

## Results

### Species Verification

The morphological similarity of closely related tuna, especially juvenile fish, can lead to species misidentification (Pedrosa-Gerasmio et al. 2012). We therefore complemented morphological species identification of our specimens with phylogenetic analyses of the mitochondrial genomes. All our specimens grouped within a monophyletic clade that included four *Thunnus albacares* mitochondrial genome sequences taken from GenBank but no sequences of other species (supplementary fig. S2, Supplementary Material online), confirming their morphological identification as *Thunnus albacares*.

### Genetic Differentiation

Pairwise fixation indices (*F*_ST_) estimated according to Weir & Cockerham (*F*_ST(W&C)_,1984) and Nei (*F*_ST(Nei)_,1987) are highly correlated (linear model; *R*^2^ = 0.976, *P* < 0.001), with Weir & Cockerham’s *F*_ST_ showing marginally larger estimates in most comparisons (mean *F*_ST(W&C)_ 0.0081; *F*_ST(Nei)_ 0.0075). Weir & Cockerham’s *F*_ST_ might be upwardly biased with small samples sizes, we therefore discuss only Nei’s *F*_ST_ in the following section, but provide both results in the supplementary table S2, Supplementary Material online. *F*_ST_ estimates indicate genetic differentiation between the Eastern North Atlantic (CAP, IVO) and the western Indo-Pacific (IND, JAP) with *F*_ST_ > 0.01 for all comparisons. In contrast, intraoceanic *F*_ST_ estimates are considerably lower (e.g., CAP vs. IVO: *F*_ST_ = 0.0034; JAP vs. IND: *F*_ST_ = 0.0033). The overall lowest *F*_ST_ estimates are found between SOU and JAP (*F*_ST_ = 0.0023) and between ELS and either USA, OMA or IND (*F*_ST_ = 0.0020, 0.0007, and −0.0004, respectively). However, low sample sizes for USA, IND, and ELS may comprise the reliability of some of these estimates, also reflected by nonsignificant *P* values (supplementary table S2, Supplementary Material online). Surprisingly, samples from the Arabian Sea (OMA) are differentiated from all other sampling sites (*F*_ST_ between 0.0084 and 0.0128) except for the sparsely sampled ELS. The overall observed heterozygosity is high (*H*_O_ = 0.45 ± 0.02) and per population *F*_IS_ estimates are negative, which could indicate outbreeding, but could also be caused by selection, associative overdominance, or differential variance in reproductive success. Alternatively, the excess of heterozygous sites could result from reads mapping to duplicated regions, despite our filtering of SNPs based on mapping quality and read depth. To minimize the inclusion of SNPs potentially affected by such reads, we applied tighter thresholds on maximum read depth (<20) and the significance level for heterozygote excess (<0.01) in separate analyses. However, these stricter criteria had only minor effects on the observed heterozygosity and the *F*_IS_ estimates (supplementary table S4, Supplementary Material online).

In the PCA, the first principal component axis (PC1) explains 3.5% of the total genomic variation, and 3.0% are explained by PC2 (fig. 2*a*). Significant separation (ANOVA, *F*_1,30_ = 96.56, *P* < 0.001) on PC1 was identified between the combined Atlantic sampling sites (USA, CAP, IVO) and the combined Pacific sampling site (JAP, IND, ELS). Samples from the Arabian Sea (OMA) are further significantly differentiated from all other samples along PC2 (ANOVA, *F*_1,42_ = 28.33, *P* < 0.001), while the IND specimens sampled near the divide between the Indian and Pacific Oceans group with the JAP samples, and are not differentiated from the Pacific sites (JAP, ELS vs. IND: ANOVA, PC1 *F*_1,16_ = 0.35, *P* = 0.57; PC2 *F*_1,16_ = 0.77, *P* = 0.35). In contrast, SOU individuals are located between the genetic clusters formed by specimens from the Atlantic and Pacific Oceans on PC1 and are significantly separated from both (SOU vs. Atlantic (USA, CAP, IVO): ANOVA, *F*_1,21_ = 49.92, *P* < 0.001; SOU vs. Pacific (IND, JAP, ELS): ANOVA, *F*_1,23_ = 5.99, *P* < 0.05). The two individuals sampled in the central-eastern Pacific (ELS) are located in different positions: one clusters within the OMA group while the other appears close to the Pacific group.

The PCA summarizes the dominant components of variation in genomic data, showing the difference between sampling sites but also including the variation within groups of samples, thus limiting the amount of between-population variation explained by the first two principal component axes. In contrast, DAPC maximizes variation between groups while minimizing within-group variation, allowing a better discrimination of predefined groups (Jombart et al. 2010). In the performed DAPC, PC1 separates Atlantic and Indo-Pacific samples and explains 73.2% of the total between-group variation while 20.2% of the variation was explained by PC2 (fig. 2*b*). Further intraoceanic differentiation between the eastern and the western Atlantic, and within the Indo-Pacific between JAP, IND, SOU, and OMA is recognizable on PC2. In contrast to the PCA, the SOU samples are included in one well-defined cluster together with the JAP and IND samples in the DAPC plot, indicating a strong Indo-Pacific influence around the Cape of Good Hope. To gain further insight into 1) intraoceanic Atlantic differentiation, and 2) the position of SOU samples, we performed two independent DAPCs including either only Atlantic, or only JAP, IND, and SOU samples, respectively. Within the intra Atlantic DAPC, East and West Atlantic sampling sites are separated on PC1 without overlap while no such separation was detected among the Indo-Pacific and SOU samples (supplementary fig. S3*a* and S3*b*, Supplementary Material online).

Multivariate analyses like PCA and DAPC describe the largest variation of the data, but they do not take advantage of population genetic models (Patterson et al. 2006). Thus, the yellowfin tuna population structure was further examined using the Bayesian model-based clustering approach implemented in the software structure. The highest likelihood value was found for *K *= 2 (fig. 2*c*, and supplementary fig. S4*a*, supplementary table S5, Supplementary Material online), which is further supported as the best fit number of genetic clusters by Evanno’s Δ*K*, an ad hoc quantity based on the rate of change of the likelihood (supplementary fig. S4*b*, Supplementary Material online; Evanno et al. 2005). According to the admixture proportions in a two-population scenario, one cluster is formed by the Eastern Atlantic samples CAP and IVO together with the OMA samples, and another cluster is formed by the western Pacific specimens of locations JAP and IND (fig. 2*c*). Samples from USA, SOU, and ELS show mixed ancestry. The average cluster membership over all SOU individuals indicates a greater similarity with Pacific than with Atlantic specimens (0.61% vs. 0.39%), in agreement with the results of the multivariate analyses. The existence of a third cluster (*K *= 3) is not apparent in the admixture plot (fig. 2*d*) and not supported by Evanno’s Δ*K* (supplementary fig. S4*b*, Supplementary Material online). Applying a sampling location prior (Hubisz et al. 2009) to assist clustering in structure did not improve cluster assignment (data not shown). Subsampling populations to an even sample number to overcome putative sample size effects (Puechmaille 2016) was performed by including only five individuals each from the Atlantic (CAP), the Pacific (JAP), the Arabian Sea (OMA), and the Pacific-Atlantic divide off South Africa (SOU) in the structure analysis, but per-population admixture proportions in the subsampled analysis are similar to those estimated in analyses with all samples, and *K* = 2 is supported by a marked increase in likelihood values (supplementary fig. S3*c* and S3*d*, and supplementary table S5, Supplementary Material online).

Since multivariate and Bayesian clustering analyses are constrained either computationally (in the case of structure), or by the method (in the case of PCA; Clavel et al. 2014), we additionally inferred the yellowfin tuna population structure using maximum-likelihood phylogenetic clustering with a less strictly filtered dataset. In agreement with the multivariate and Bayesian clustering methods, the maximum-likelihood tree shows two well-separated clades, of which one is composed of individuals from the Indo-Pacific (JAP, IND) and the other includes all Atlantic (USA, CAP, IVO) specimens (fig. 2*e*). All but one of the OMA samples are nested within the Atlantic clade, supporting the cluster assignment of the structure analysis. The single OMA individual sharing high ancestry with the Indo-Pacific cluster in the structure analysis also appears within the Indo-Pacific clade in the phylogenetic tree. Therefore, the OMA clade can be viewed as a nearly monophyletic group and may represent a distinct genetic cluster. Furthermore, all except one SOU individual are located in a single clade that groups with the Indo-Pacific clade, in agreement with the results of the structure and multivariate analyses. However, bootstrap support for most branches is weak (<80) and thus these groups should be interpreted with caution (supplementary fig. S3*e*, Supplementary Material online). Given the genetic differentiation of Atlantic or Indo-Pacific yellowfin tuna, we were also able to identify a minimal panel of ten SNPs allowing the assignment of individuals to their origins with an accuracy of 91%, although the small sample size necessitates further validation (data therefore not shown).

**Fig. 3.—:**
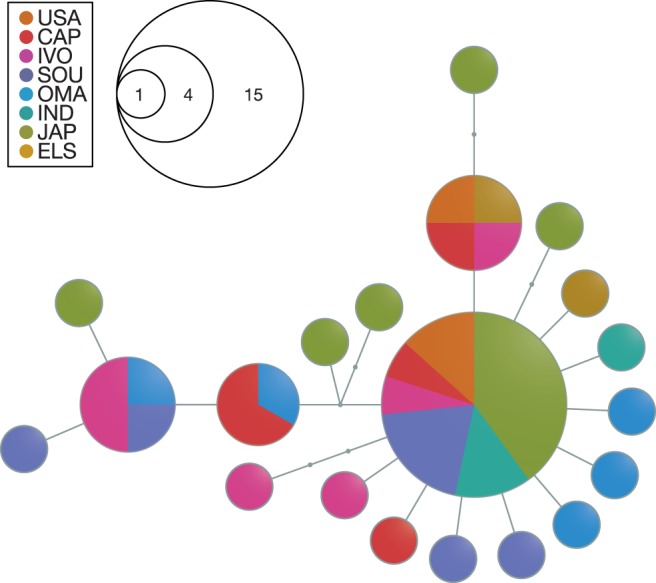
Unrooted haplotype genealogy graph of yellowfin tuna mitochondrial genomes. Edge lengths correspond to the number of transversions (total 25), and node sizes are proportional to haplotype frequencies. Sampling sites for haplotypes are indicated by node fragment colors.

In addition to nuclear SNPs, we also investigated the genetic relationships of yellowfin tuna based on haplotypes derived from full mitochondrial genomes (fig. 3). The mitochondrial alignment resulted in a total of 409 variable sites (2.48%), of which a maximum-parsimony tree with a minimum of 583 substitutions was constructed. Mitochondrial genetic variation was illustrated as a haplotype genealogy graph based on the maximum-parsimony tree, with graph edge lengths calculated from transversions only. The haplotype genealogy graph includes 20 nodes, each representing one or more sampled mitochondrial sequences that are identical or separated only by transitions. To connect these 20 sampled nodes, a total of 25 transversions were required (fig. 3), indicating incomplete sampling of the genetic diversity presumably due to low sample sizes, as six intermediate nodes were inferred for which no sequences were present in our dataset. One major central node represents sequences of 15 individuals from all locations except OMA and ELS, however, individuals from OMA and ELS are separated by only a single transversion from this central node. In addition, three nodes represent sequences sampled from more than one individual, and in these cases, the sequences are found both in Atlantic and Indo-Pacific specimens.

### Demography and Gene Flow

**Fig. 4.—:**
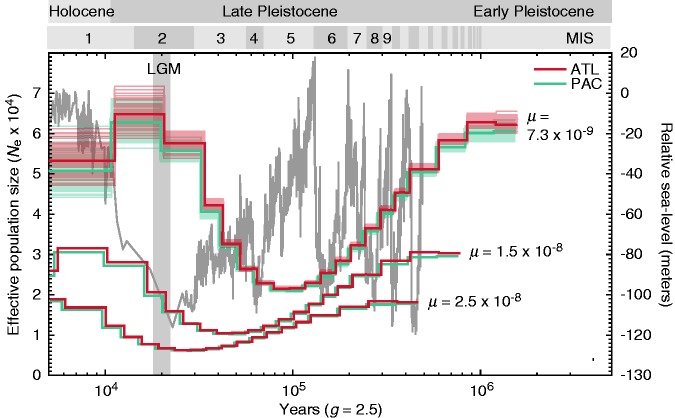
Yellowfin tuna demographic history. Effective population size (*N*_e_, left vertical axis) changes estimated with pairwise sequential Markovian coalescent analysis (PSMC) of one Atlantic (ATL, red) and one Indo-Pacific (PAC, blue) yellowfin tuna individual genome sequence over the last 1 myr (horizontal axis). Estimates obtained for three different mutation rates are shown (*μ* = 7.3 × 10^−9^, 1.5 × 10^−8^, and 2.5 × 10^−8^ mutations/site/generation). Semitransparent colored lines correspond to confidence intervals derived from 100 bootstrap replicates shown only for *μ* = 7.3 × 10^−9^ to increase visibility. Historic global relative sea-level fluctuations are indicated as gray line (right vertical axis), adapted from Grant et al. (2014). MIS, marine isotope stages, LGM (Last Glacial Maximum).

Models of demographic history can help to identify population differentiation by inferring past population-specific demographic changes. We used the pairwise sequential Markovian coalescent (PSMC; Li and Durbin 2011) on single representatives of the Atlantic (ATL) and Indo-Pacific (PAC) Oceans to infer the demographic history of the two populations. Both genomes feature a high number of heterozygous sites (3.2 × 10^6^), indicating high overall genetic variability. With an assumed mutation rate of 7.3 × 10^−9^ mutations/site/generation, the PSMC estimates of effective population sizes (*N*_e_; fig. 4) individually fluctuate between ∼20,000 and ∼65,000, and demonstrate severe reductions in population size in the Late Pleistocene, followed by population expansions towards the Holocene and another decrease near recent times. Both the ATL and PAC genomes indicate similar trajectories over the last 1 myr with population expansions and bottlenecks roughly following the fluctuations in relative sea level in an anticyclical pattern, showing a decline in population size at higher sea levels during marine isotope stages (MIS) 6 to ∼20, and an expansion of population size during low sea levels in the glacial periods corresponding to MIS 2–4. When applying higher alternative mutation rates (1.5 × 10^−8^ and 2.5 × 10^−8^ mutations/site/generation) to account for PSMC parameter uncertainties, the inferred population size changes are shifted towards more recent times and the overall estimates of *N*_e_ are lowered to fluctuate between ∼5,000 and ∼30,000, with the lowest estimates corresponding to a bottleneck around the time of the Last Glacial Maximum (LGM, ∼21 ka; fig. 4).

Demographic parameters were also estimated through coalescent simulations with fastsimcoal2, comparing simulated SFS with the observed joint SFS of Atlantic and Indo-Pacific yellowfin tuna populations. Using the best-fitting ten out of 80 replicate sets of analyses, the effective population sizes of the ancestral, the Atlantic, and the Indo-Pacific yellowfin tuna populations were estimated as 33,122.0 ± 16,018.9, 1,955.2 ± 625.6, and 52,423.2 ± 20,733.6, respectively (mean across ten replicates ± standard deviation). The divergence time between Atlantic and Indo-Pacific yellowfin tuna was estimated as 33,564.3 ± 7,603.4 generations, and the migration rates were estimated as 0.048 ± 0.014 from the Indo-Pacific to the Atlantic, and 0.003 ± 0.003 in the opposite direction, from the Atlantic to the Indo-Pacific. Qualitatively similar estimates were obtained when removing the heterogenic ELS sample from the Indo-Pacific population (supplementary table S6, Supplementary Material online).

## Discussion

### Yellowfin Tuna is Genetically Differentiated between Major Oceans

By using a range of discriminative methods, we thoroughly investigated the global population structure of yellowfin tuna and found a clear separation between Atlantic and Indo-Pacific groups as well as indicative evidence for a third genotypic cluster within the Arabian Sea. This largely confirms recent preliminary results based on reduced-representation genome sequencing, which also pointed at separation between oceans (Pecoraro et al. 2015). However, the existence of yellowfin tuna population structure has been controversial, as long-distance movements are known to occur in this highly migratory species (Itano and Williams 1992; Ortiz 2001; Fonteneau and Hallier 2015) which could potentially lead to a homogenization of between-ocean genetic differentiation. The distribution of yellowfin tuna is restricted to tropical and subtropical regions except the Mediterranean; therefore dispersal between the Atlantic and Indo-Pacific Oceans is only possible to the south of Africa around the Cape of Good Hope. However, this route may not be favorable for the migration of warm-water fishes as it would involve the crossing of the strong Benguela Current, which transports cold upwelled water northward along the southwest coast of Africa and is a potential barrier to gene flow (Henriques et al. 2014). Moreover, even though extensive tagging programs have been implemented for tropical tunas (e.g., Fonteneau and Hallier 2015), we did not find tagging-related literature that directly demonstrates dispersal between the Atlantic and Indo-Pacific Oceans. Previous genetic analyses based on mitochondrial sequences showed a strong separation of Atlantic and Indo-Pacific populations of the closely related bigeye tuna (*Thunnus obesus*) with an admixture zone off South Africa (Chow et al. 2000; Durand et al. 2005; Gonzalez et al. 2008). On the other hand, a subsequent investigation found no evidence of differentiation based on nuclear DNA and the authors attributed the incongruence of mitochondrial and nuclear patterns to separation during glacial maxima followed by secondary contact (Gonzalez et al. 2008). Using nuclear genomic SNPs, we detected a clear differentiation between Atlantic and Indo-Pacific yellowfin tuna in this study. Unlike in bigeye tuna (Gonzalez et al. 2008), mitochondrial yellowfin tuna sequences alone did not support separate Atlantic and Indo-Pacific clades. A lack of mitochondrial separation was also previously found based on D-loop sequences (Ely et al. 2005). In contrast, RFLP analysis of a slower-evolving mitochondrial gene indicated low levels of genetic differentiation, leading the authors to suggest that homoplasy and high levels of haplotypic diversity would mask differentiation in the rapidly evolving D-loop (Ely et al. 2005). However, our analysis of the mitochondrial genome excluding the D-loop resulted in a haplotype genealogy that was qualitatively very similar to the one based on full mitochondrial genomes and also did not show any separation between Atlantic and Indo-Pacific populations (data not shown). Thus, the disagreement between the identified population structure in the mitochondrial RFLP data (Ely et al. 2005) and the lack of structure in the mitochondrial genome sequences produced in our study could be attributed to either stochastic effects in the RFLP analysis, or to insufficient sampling of individuals in our analysis. In addition, the fact that we detected population separation with a much larger nuclear dataset suggests that our mitochondrial dataset and possibly those used in previous studies may suffer a lack of power to detect population differentiation. Alternatively, the discordance between nuclear and mitochondrial patterns could also arise by predominantly female migration and dispersal. Genetic differentiation between Atlantic and Indo-Pacific groups has also been described for two other circumtropical members of the genus *Thunnus*: albacore (*T. alalunga*; Albaina et al. 2013; Laconcha et al. 2015) and bigeye tuna (*T. obesus*; Chow et al. 2000; Martínez et al. 2006; Gonzalez et al. 2008). However, using traditional genetic tools, a single population was found for the southern bluefin tuna (*T. maccoyii*) with a circumglobal distribution in the temperate waters of the southern Atlantic and Indo-Pacific (Grewe et al. 1997), and for the closest relative of the genus *Thunnus*, the skipjack tuna (*Katsuwonus pelamis*; Graves et al. 1984; Ely et al. 2005). It remains to be investigated whether increased resolution provided by genomic datasets will also be able to decipher population genetic structuring in these species.

In contrast to the marked differentiation of Atlantic and Indo-Pacific yellowfin tuna based on our nuclear SNP dataset, genetic separation between the Indian Ocean and the Pacific is less pronounced, indicating that larger sample sizes might be necessary to identify differentiation between these regions. Our Indonesian samples cluster with northwestern Pacific samples in all analyses, pointing towards a major influence of the Pacific to the waters around Indonesia. The Indonesian Throughflow, a strong ocean current running from the Pacific to the Indian Ocean through a series of narrow straits between the Indonesian islands, transports warm Pacific water into the eastern Indian Ocean (fig. 1; Sprintall et al. 2014) and may facilitate dispersal between these water bodies. Other studies on circumtropical tunas also found little or no genetic heterogeneity between Indo-Pacific populations but identified genetic differentiation within the Atlantic (Gonzalez et al. 2008; Laconcha et al. 2015). This trend is apparent in our intraoceanic analyses, indicating differentiation between the western and eastern Atlantic, but not between South Africa, Indonesia, and Japan. Nevertheless, our samples from the northern Indian Ocean (Arabian Sea) are genetically differentiated from both the Atlantic and the Indo-Pacific (IND, JAP) clusters in multivariate and phylogenetic analyses. Morphological differences exist between yellowfin tuna from the Indian Ocean off Somalia and other Atlantic and Pacific samples (Royce 1964); furthermore, previous analyses of yellowfin tuna mitochondrial DNA on a smaller geographic scale showed differentiated groups in the northeastern and southeastern Arabian Sea and also in the Bay of Bengal (Kunal et al. 2013). Concordantly, our mitochondrial haplotype genealogy graph shows only two nodes that are shared between samples from the Oman and other areas, which may indicate genetic isolation of the Arabian Sea population. In addition, tagging data for Indian Ocean yellowfin tuna suggest largely restricted movements within the western Indian Ocean (IOTC 2015). Recent preliminary analyses utilizing reduced-representation genome sequencing showed differentiated clusters of Atlantic and Pacific yellowfin tuna, with a third genetically distinct cluster in the western Indian Ocean (between ∼0° and 10°S latitude; Pecoraro et al. 2015) that appeared to share more ancestry with the Pacific than the Atlantic cluster. In contrast, our samples from the Arabian Sea in the northern Indian Ocean show greater similarity with the Atlantic clade in both the admixture and phylogenetic analysis, and also share mitochondrial haplotypes with Atlantic samples. The Arabian Sea in the northern Indian Ocean is influenced by strong seasonal monsoon cycles with increased productivity (Singh et al. 2011), where yellowfin tuna populations have one major reproductive season during the winter monsoon (November–February; Stequert et al. 2001). This is contrary to other populations; for example, in the western Pacific spawning occurs all year with a peak between February and June (Sun et al. 2005) and in the northwestern Atlantic, where yellowfin tuna spawn between March and November (Arocha et al. 2001). Thus, discrete spawning times in the Arabian Sea could potentially have driven genetic divergence. High fishing pressure constitutes a major problem in the Arabian Sea and the most recent stock-size estimates indicate overfishing of the Indian ocean yellowfin tuna as a result of large and unsustainable catches (IOTC 2015). Follow-up studies with larger sample sizes will therefore be of high priority to delineate the existence of a genetically and biologically diverged yellowfin tuna population in this area.

Intraoceanic differentiation has been described between the western, central, and eastern Pacific (Ward et al. 1994; Grewe et al. 2015). In our analysis, we included only two samples from the central-eastern Pacific. Although the low sample size does not allow detailed conclusions, the genetic heterogeneity of these samples suggests a highly structured Pacific population as shown previously, or alternatively may be due to migratory individuals. More extensive sampling of individuals would be needed for further investigations of population structure within the Pacific.

### Demographic Histories and Asymmetric Gene Flow between Atlantic and Indo-Pacific Populations

Genomes do not only convey information about current population structure, but also hold clues to past demographic change and can therefore provide information about the evolutionary history of populations. Methods for the inference of these demographic histories, such as PSMC or coalescent simulations, require estimates of the genome-wide mutation rate, which have so far not been described for yellowfin tuna. Therefore, we calculated a mutation rate of yellowfin tuna based on a genomic comparison with the closely related bluefin tuna, for which divergence time estimates are available. Using the resulting mutation rate estimate of 7.3 × 10^−9^ mutations/site/generation, our PSMC analyses recovered a past population expansion that began around 80 ka with an *N*_e_ around 20,000 and ended about 20 ka when the *N*_e_ was around 65,000. However, as the effective within-population mutation rates are often higher than those observed between species, the use of a phylogenetically derived mutation rate may lead to overestimation of the timing of past population size changes (Ho et al. 2005). To compensate for this potential bias, we therefore ran additional sets of PSMC analyses with higher mutation rate estimates, adopting the human mutation rate (2.5 × 10^−8^ mutations/site/generation) as well as an intermediate rate estimate (1.5 × 10^−8^ mutations/site/generation). With these rates, the population expansion appears more recent, and effective population size estimates are also lower than with the phylogenetically derived rate. The demographic history, particularly when estimated using the faster human mutation rate, appears to be influenced by climatic changes related to Pleistocene glaciation cycles, as shown previously using the same method for the killer whale (Moura et al. 2014), but also for another member of the family Scombridae (the Pacific Sierra mackerel) using mismatch distributions (López et al. 2010). Earlier estimates of the past demography of yellowfin tuna based on mitochondrial D-loop mismatch distributions suggested a very high *N*_e_ of ten million subsequent to a population expansion about 500 ka (Ely et al. 2005). In contrast, our PSMC estimates using the phylogenetically derived mutation rate (7.3 × 10^−9^) suggest a population size expansion between 80 and 20 ka with an *N*_e_ of ∼65,000 after the expansion. The great discrepancy of the population size estimates could result from stochasticity affecting one or both of the datasets, from the different models used to estimate demographic parameters, or from different assumptions made for model parameters such as the mutation rate and the generation time. Ely and coauthors assumed a generation time of 3.5 years, whereas we assumed a shorter time of 2.5 years according to IUCN estimates (Collette et al. 2011). However, the use of a generation time of 3.5 years in combination with adjusted mutation rate estimates (1.0 × 10^−8^ mutations/site/generation) does not change the shape or the timing of the PSMC demographic curve, but instead leads to even lower estimates of *N*_e_ (between 15,000 and 45,000; data not shown). Similarly, the use of alternative mutation rates in PSMC analyses also decreases the estimates of *N*_e_. Therefore, it is likely that the large difference between population size estimates in our study and Ely et al. (2005) is due to either an underestimation of the mitochondrial mutation rate in Ely et al. (2005) or differences between the applied models. Furthermore, we cannot exclude the possibility that our PSMC results might be influenced by the inclusion of sex-determinating regions, since these have not yet been identified in tunas. By also estimating demographic parameters with coalescent simulations in fastsimcoal2, we complemented our PSMC analyses, which allow gradual changes in population size over time, with a model in which population sizes are constant except at a single divergence time. In agreement with PSMC, estimates based on this model indicated effective population sizes in the ancestral and the Indo-Pacific population on the order of a few ten thousands (ancestral *N*_e_: 33122.0 ± 16018.9, Indo-Pacific *N*_e_: 52423.2 ± 20733.6), thus corroborating that the true values of *N*_e_ are far below those estimated in Ely et al. (2005).

All coalescent-based analyses used in our study, as well as the mismatch analysis used in Ely et al. (2005) share assumptions that may be violated in the investigated populations, which could lead to biased estimates of demographic parameters. These assumptions include a bifurcating genealogy, which may be inappropriate in highly fecund marine species with large differences in reproductive success between individuals. Such differences in reproductive success can cause overestimates of the timing of population expansion as well as *N*_e_ (Eldon and Wakeley 2006; Grant 2016).

Furthermore, coalescent-based demographic analyses are based on the assumption of panmictic populations, which can lead to bias and overestimation of *N*_e_ at the time of population subdivision if population structure is present (Li and Durbin 2011). Thus, an unknown split of yellowfin tuna populations during the LGM into separate refugia, followed by secondary contact could induce temporally elevated estimates of *N*_e_, such as those seen in our analysis. Repeated glacial cycles and associated habitat changes in the Pleistocene have influenced the distribution and abundance of many species (Hewitt 2004) including tropical marine fish (Ludt and Rocha 2014), and have had genomic consequences for these species due to population bottlenecks and expansions. However, to what extent glacial cycles, and thus changing sea levels, temperature, ocean currents, and oceanic productivity have influenced highly mobile tropical species like the yellowfin tuna remains unknown.

Our PSMC analyses indicate similar demographic histories in Atlantic and Indo-Pacific yellowfin tuna specimens. This similarity could be explained if population size changes in both oceans were in fact driven by the same common factors, such as sea level changes or climatic fluctuations. Alternatively, similar demographic trajectories could result if the two populations diverged only recently, or if they are connected by substantial genetic exchange that led to a homogenization of the genetic information used to infer demography. Such connectivity between the Atlantic and Indo-Pacific is supported by the results of our simulation-based estimation of demographic parameters with fastsimcoal2, indicating that migration across the Benguela Current (see fig. 1) is possible for warm-water fishes like yellowfin tuna. However, migration rate estimates were about ten times higher from the Indo-Pacific to the Atlantic than in the opposite direction, which suggests that gene flow across the Benguela Current is largely unidirectional. This dispersal could be facilitated by the warm Agulhas Current, which transports tropical water from the Indian Ocean along the west African continental shelf up to the Cape of Good Hope, where it mostly retroflects back towards the Indian Ocean. Occasionally, warm-water eddies detach from the Agulhas Current south of South Africa (so-called “Agulhas rings;” Schouten et al. 2000), some of which penetrate the Benguela Current and subsequently merge with warmer waters of the Atlantic (Duncombe Rae 1991). Thus, Agulhas rings increase dispersal potential for warm-water species into the Atlantic, and could thus be the cause for the observed directionality of gene flow in yellowfin tuna, as well as other circumtropically distributed fishes, including the bigeye tuna (Chow et al. 2000; Durand et al. 2005; Gonzalez et al. 2008; Gaither et al. 2016).

### Summary and Significance of the Study

We here provide strong evidence for genetic differentiation between Atlantic and Indo-Pacific yellowfin tuna populations, as well as indications for further divergence within the Arabian Sea. The inference of population structure in highly migratory marine organism like the yellowfin tuna has proven to be a difficult task, which can cause pivotal adaptive variation to remain overlooked, thus risking the overexploitation and extinction of locally adapted populations, or even species. Abundance and distribution of tuna have been connected with climatic changes and weather oscillations (Kumar et al. 2014; Dueri et al. 2014), and the ongoing ocean warming may thus lead to range shifts in some populations. The implementation of genetic population structure information into conservation can thus help to preserve genetic diversity, securing a healthy ecosystem and sustainable fisheries for the future.

## Supplementary Material

Supplementary data are available at *Genome Biology and Evolution* online.

## Author Contributions

The study was conceived and designed by R.H., S.J. and M.D. with contributions from J.M.I.B. DNA was extracted by J.M.I.B., and sequencing libraries were generated by J.M.I.B. and M.D. The data was analyzed by J.M.I.B. with contributions from M.M. and M.D. The manuscript was written by J.M.I.B. and M.D. with contributions from all authors.

## Supplementary Material

Supplementary Data
