# Supplementary material for: Genomic Differentiation and Demographic Histories of Atlantic and Indo-Pacific Yellowfin Tuna (Thunnus albacares) Populations
Source: Genome Biol Evol. 2017 Apr 1;9(4):1084–98. doi: 10.1093/gbe/evx067 (PMC5408087; doi:10.1093/gbe/evx067)
Supplement: Supplementary Data [file evx067_Supp.pdf]

Supporting information "Genomic differentiation and demographic histories of Atlantic and Indo-Pacific yellowfin tuna (*Thunnus albacares*) populations"

Figure S1 Linkage disequilibrium decay.

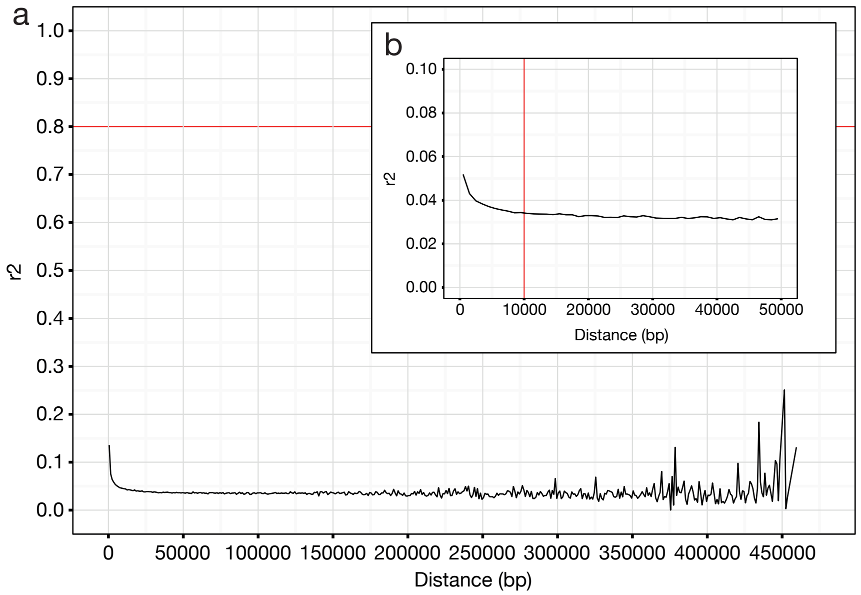

Fig. S1 Linkage disequilibrium decay and filtering thresholds. (a) Linkage disequilibrium measured by the mean squared correlation coefficient (r^2) in 1 kb bins, as a function of the genetic distance between markers (bp). (b) Detail view of (a) for marker distances up to 50,000 bp. The threshold for the exclusion of highly linked SNPs was set as $r^2 > 0.8$ for markers separated by less than 10 kb (see red lines in (a) and (b)).

Figure S2 Species identification.

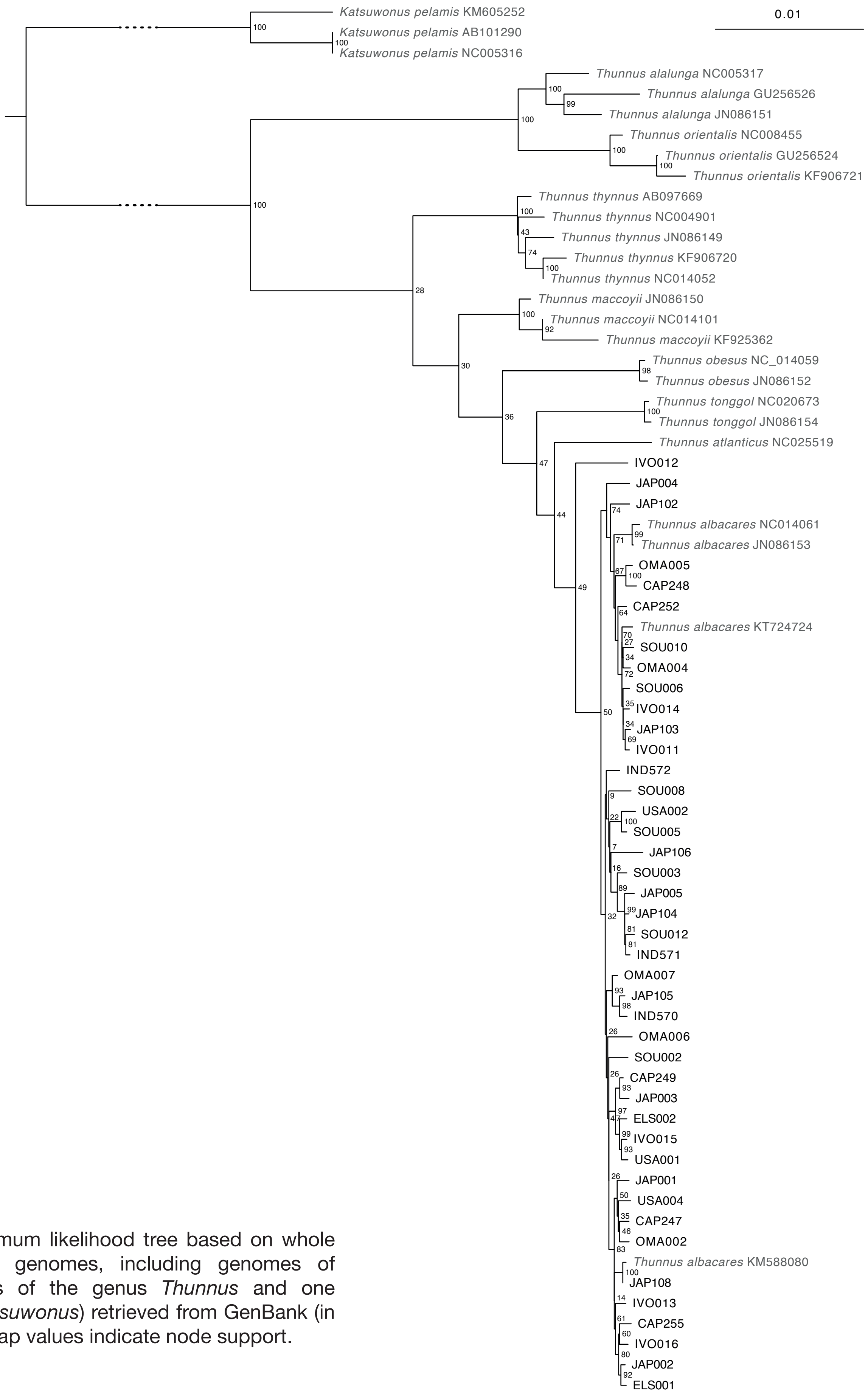

Fig. S2 Maximum likelihood tree based on whole mitochondrial genomes, including genomes of eight species of the genus *Thunnus* and one outgroup (*Katsuwonus*) retrieved from GenBank (in gray). Bootstrap values indicate node support.

Figure S3 Genetic differentiation of yellowfin tuna.

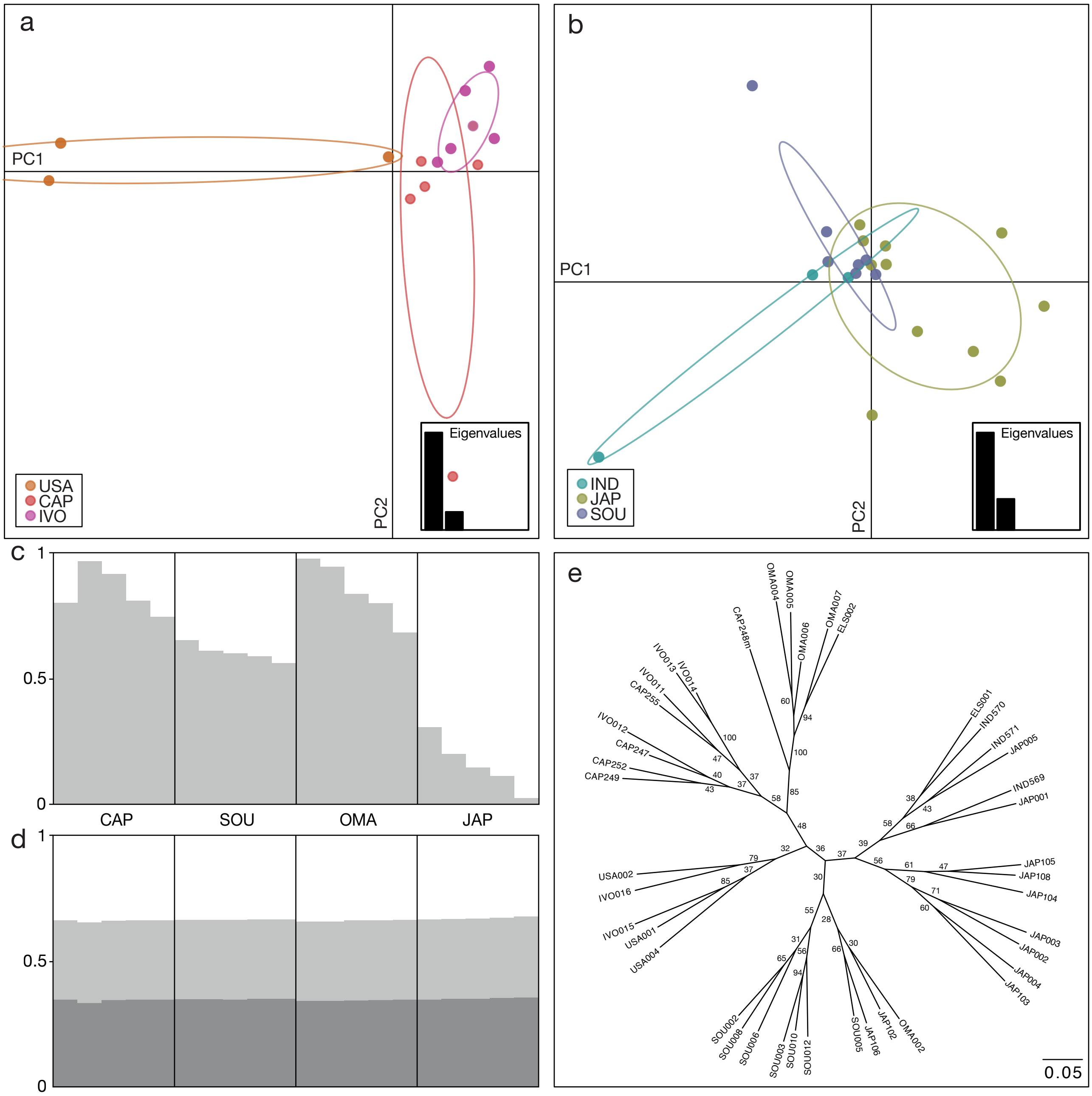

Fig. S3 Genetic differentiation of yellowfin tuna. (a,b) Discriminant analysis of principal components (DAPC) describing the variation between sampling sites within the Atlantic (a) and Indo-Pacific (b). Inertia ellipses summarize the variation per sampling site and eigenvalues for the first five PCs are displayed in bar plot insets (those for PC3-PC5 are too small to display). (c,d) Individual admixture bar plots of STRUCTURE analysis using an even sample size of 5 individuals per sampling site. STRUCTURE *q*-values (vertical axis) are shaded according to cluster membership, black bars separate sampling locations. Within sampling sites, individuals are sorted according to assignment proportions. Number of tested clusters: (c) $K = 2$, (d) $K = 3$. (e) Maximum-likelihood tree as in Fig. 2e including sample IDs and bootstrap values based on 100 replicates. The scale bar indicates the number of substitutions.

Figure S4 STRUCTURE likelihood values.

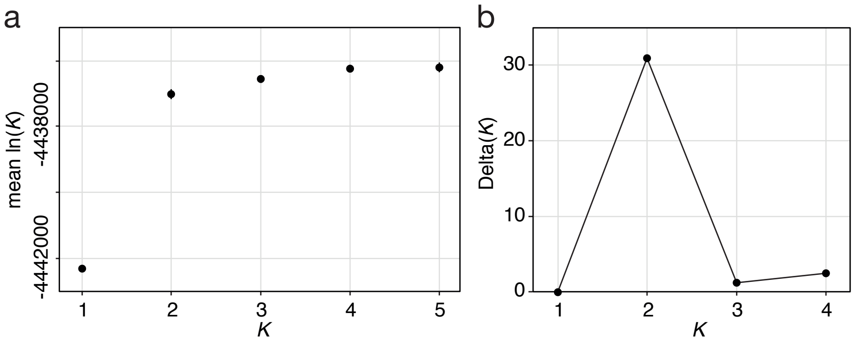

Fig. S4 STRUCTURE likelihood values (a) and Δ*K* (Evanno et al., 2005) for the analysis including all individuals (see Fig. 2c,d) with *K* = 1 to *K* = 5. (a) Mean likelihood (ln) at each *K* including standard deviation over 5 repetitions. (b) Δ *K* for each *K*.

Table S1 Overview of yellowfin tuna samples. Sample ID, sampling location, and sampling date. FAO, Food and Agriculture Organization of the United Nations.

Sample ID	Sampling location		Sampling date
	FAO fishing area	Location	
USA001	Atlantic, Northwest	U.S.A., Rhode Island, local fish market	2014, June
USA002	Atlantic, Northwest	U.S.A., Rhode Island, local fish market	2014, Sept.
USA004	Atlantic, Northwest	U.S.A., Rhode Island, local fish market	2014, Sept.
CAP247	Atlantic, Eastern Central	Republic of Cabo Verde, Mindelo	2014
CAP248	Atlantic, Eastern Central	Republic of Cabo Verde, Mindelo	2014
CAP249	Atlantic, Eastern Central	Republic of Cabo Verde, Mindelo	2014
CAP252	Atlantic, Eastern Central	Republic of Cabo Verde, Mindelo	2014
CAP255	Atlantic, Eastern Central	Republic of Cabo Verde, Mindelo	2014
IVO011	Atlantic, Eastern Central	Ivory Coast, Abidjan	2014, May
IVO012	Atlantic, Eastern Central	Ivory Coast, Abidjan	2014, May
IVO013	Atlantic, Eastern Central	Ivory Coast, Abidjan	2014, May
IVO014	Atlantic, Eastern Central	Ivory Coast, Abidjan	2014, May
IVO015	Atlantic, Eastern Central	Ivory Coast, Abidjan	2014, May
IVO016	Atlantic, Eastern Central	Ivory Coast, Abidjan	2014, May
SOU002	Atlantic, Southeast	South Africa, Cape Town, 34° S, 18° E	2014, May
SOU003	Atlantic, Southeast	South Africa, Cape Town, 34° S, 18° E	2014, May
SOU005	Atlantic, Southeast	South Africa, Cape Town, 34° S, 18° E	2014, May
SOU006	Atlantic, Southeast	South Africa, Cape Town, 34° S, 18° E	2014, May
SOU008	Atlantic, Southeast	South Africa, Cape Town, 34° S, 18° E	2014, May
SOU010	Atlantic, Southeast	South Africa, Cape Town, 34° S, 18° E	2014, May
SOU012	Atlantic, Southeast	South Africa, Cape Town, 34° S, 18° E	2014, May
IND570	Indian Ocean, Eastern	Indonesia, Denpasar, 10°S, 110°E	2012, July
IND571	Indian Ocean, Eastern	Indonesia, Denpasar, 10°S, 110°E	2012, July
IND572	Indian Ocean, Eastern	Indonesia, Denpasar, 10°S, 110°E	2012, July
OMA002	Indian Ocean, Western	Oman, Muscat, local fish market	2014, Feb.
OMA005	Indian Ocean, Western	Oman, Barka, local fish market	2014, Feb.
OMA006	Indian Ocean, Western	Oman, Barka, local fish market	2014, Feb.
OMA007	Indian Ocean, Western	Oman, Barka, local fish market	2014, Feb.
OMA004	Indian Ocean, Western	Oman, Barka, local fish market	2014, Feb.
JAP001	Pacific, Northwest	Japan, Sagami Bay	2014, July
JAP002	Pacific, Northwest	Japan, Sagami Bay	2014, July
JAP003	Pacific, Northwest	Japan, Sagami Bay	2014, July
JAP004	Pacific, Northwest	Japan, Sagami Bay	2014, July
JAP005	Pacific, Northwest	Japan, Sagami Bay	2014, July
JAP102	Pacific, Northwest	Japan, Kozu Island	2014, Aug.
JAP103	Pacific, Northwest	Japan, near Okinawa Island	2014, Aug./Sept.
JAP104	Pacific, Northwest	Japan, near Okinawa Island	2014, Aug./Sept.
JAP105	Pacific, Northwest	Japan, near Okinawa Island	2014, Aug./Sept.
JAP106	Pacific, Northwest	Japan, near Okinawa Island	2014, Aug./Sept.
JAP108	Pacific, Northwest	Japan, Kozu Island	2014, Aug.
ELS001	Pacific, Eastern Central	3° N, 154° W	2014, June
ELS002	Pacific, Eastern Central	5° N, 104° W	2014, June

Table S2 Pairwise F_{ST} according to Weir and Cockerham, 1984 (below diagonal), and Nei, 1987 (above diagonal). Significance levels for Weir and Cockerham F_{ST} only: $p < 0.05$ *; $p < 0.01$ **; $p < 0.001$ ***.

	USA	CAP	IVO	SOU	OMA	IND	JAP	ELS
**USA**	NA	0.0025	0.0045	0.0068	0.0092	0.0044	0.0046	0.0020
**CAP**	0.0030	NA	0.0034	0.0104	0.0084	0.0145	0.0126	0.0095
**IVO**	0.0054***	0.0033***	NA	0.0120	0.0128	0.0160	0.0125	0.0132
**SOU**	0.0082***	0.0105***	0.0120***	NA	0.0080	0.0053	0.0023	0.0079
**OMA**	0.0096***	0.0084***	0.0129***	0.0082***	NA	0.0109	0.0089	0.0007
**IND**	0.0045	0.0152***	0.0171***	0.0069***	0.0115***	NA	0.0033	-0.0004
**JAP**	0.0052***	0.0124***	0.0122***	0.0018**	0.0088***	0.0041***	NA	0.0034
**ELS**	0.0021	0.0108***	0.0150***	0.0103***	0.0017	-0.0003	0.0046**	NA

Table S3 Pairwise Weir and Cockerham F_{ST} for varying thresholds for missing data (10%, 20%) and minor allele frequency (no filtering for MAF (no MAF); MAF < 0.1 (MAF 0.1); MAF < 0.2 (MAF 0.2); MAF < 0.3 (MAF 0.3).

	**Max. 10% missing data**				**Max. 20% missing data**			
	**noMAF**	**MAF0.1**	**MAF0.2**	**MAF0.3**	**noMAF**	**MAF0.1**	**MAF0.2**	**MAF0.3**
**CAP-IVO**	0.0026	0.0033	0.0041	0.0046	0.0027	0.0027	0.0027	0.0027
**CAP-SOU**	0.0094	0.0105	0.0103	0.0091	0.0082	0.0087	0.0087	0.0083
**CAP-OMA**	0.0074	0.0084	0.0086	0.0070	0.0060	0.0064	0.0065	0.0062
**CAP-IND**	0.0140	0.0152	0.0151	0.0141	0.0121	0.0127	0.0133	0.0143
**CAP-JAP**	0.0109	0.0124	0.0132	0.0124	0.0096	0.0107	0.0116	0.0120
**CAP-ELS**	0.0096	0.0108	0.0115	0.0119	0.0069	0.0080	0.0088	0.0088
**CAP-USA**	0.0024	0.0030	0.0036	0.0041	0.0035	0.0045	0.0054	0.0063
**IVO-SOU**	0.0107	0.0120	0.0114	0.0101	0.0098	0.0096	0.0088	0.0077
**IVO-OMA**	0.0116	0.0129	0.0124	0.0111	0.0098	0.0103	0.0103	0.0099
**IVO-IND**	0.0160	0.0171	0.0153	0.0147	0.0130	0.0127	0.0122	0.0127
**IVO-JAP**	0.0111	0.0122	0.0123	0.0113	0.0104	0.0113	0.0120	0.0128
**IVO-ELS**	0.0141	0.0150	0.0166	0.0155	0.0112	0.0119	0.0120	0.0120
**IVO-USA**	0.0046	0.0054	0.0057	0.0058	0.0055	0.0063	0.0068	0.0077
**SOU-OMA**	0.0067	0.0082	0.0076	0.0069	0.0104	0.0112	0.0120	0.0112
**SOU-IND**	0.0056	0.0069	0.0081	0.0091	0.0051	0.0068	0.0084	0.0096
**SOU-JAP**	0.0017	0.0018	0.0018	0.0014	0.0032	0.0024	0.0017	0.0011
**SOU-ELS**	0.0082	0.0103	0.0136	0.0154	0.0065	0.0111	0.0150	0.0167
**SOU-USA**	0.0072	0.0082	0.0086	0.0092	0.0073	0.0097	0.0112	0.0124
**OMA-IND**	0.0106	0.0115	0.0117	0.0097	0.0127	0.0132	0.0140	0.0144
**OMA-JAP**	0.0076	0.0088	0.0091	0.0080	0.0097	0.0111	0.0128	0.0123
**OMA-ELS**	0.0020	0.0017	0.0013	0.0002	0.0042	0.0044	0.0048	0.0046
**OMA-USA**	0.0091	0.0096	0.0087	0.0068	0.0118	0.0130	0.0139	0.0136
**IND-JAP**	0.0041	0.0041	0.0035	0.0034	0.0028	0.0027	0.0022	0.0025
**IND-ELS**	-0.0004	-0.0003	-0.0006	-0.0004	-0.0021	-0.0020	-0.0020	-0.0026
**IND-USA**	0.0051	0.0045	0.0047	0.0034	0.0035	0.0034	0.0031	0.0027
**JAP-ELS**	0.0042	0.0046	0.0065	0.0063	0.0026	0.0042	0.0055	0.0051
**JAP-USA**	0.0048	0.0052	0.0062	0.0054	0.0051	0.0065	0.0074	0.0081
**ELS-USA**	0.0031	0.0021	0.0035	0.0021	0.0009	0.0007	0.0000	-0.0014

Table S4 Observed heterozygosity (H_O), within population gene diversity (H_S), and inbreeding coefficient (F_{IS}) for datasets filtered at different maximum read depths (DP) and heterozygosity excess significance (HEX *p*-value) thresholds.

	DP < 20, HEX < 0.01			DP < 100, HEX < 0.0001		
	H_O	H_S	F_{IS}	H_O	H_S	F_{IS}
**USA**	0.42	NaN	-0.12	0.44	NaN	-0.18
**CAP**	0.43	0.38	-0.12	0.45	NaN	-0.17
**IVO**	0.42	0.38	-0.11	0.44	0.37	-0.16
**SOU**	0.48	0.40	-0.19	0.48	0.39	-0.22
**OMA**	0.42	0.38	-0.11	0.44	0.37	-0.16
**IND**	0.44	NaN	-0.15	NaN	NaN	-0.20
**JAP**	0.43	0.39	-0.11	0.44	0.38	-0.16
**ELS**	NaN	NaN	-0.16	NaN	NaN	-0.21

Table S5 STRUCTURE mean ln likelihood values for replicate (Rep.) 1 – 5 and *K* 1 – 5.

A) All individuals, see Fig. 2c,d.

	**Mean ln likelihood**				
K	**Rep. 1**	**Rep. 2**	**Rep. 3**	**Rep. 4**	**Rep. 5**
**1**	-4442321.0	-4442321.8	-4442320.7	-4442319.8	-4442319.8
**2**	-4436890.5	-4437153.3	-4436804.8	-4437091.5	-4437131.0
**3**	-4436422.8	-4436702.4	-4436435.7	-4436627.2	-4436608.2
**4**	-4436156.0	-4436129.7	-4436387.8	-4436208.8	-4436346.7
**5**	-4436030.1	-4436349.3	-4436164.6	-4436145.0	-4436369.2

B) Even sampling (5 individuals per sampling site), see Fig. S2c,d.

	**Mean ln likelihood**				
K	**Rep. 1**	**Rep. 2**	**Rep. 3**	**Rep. 4**	**Rep. 5**
**1**	-2116002.5	-2116000.8	-2116001.7	-2116000.7	-2116002.0
**2**	-2114837.7	-2114871.0	-2114827.7	-2114909.0	-2114913.6
**3**	-2115926.1	-2115924.8	-2115927.9	-2115925.2	-2115927.5
**4**	-2115912.7	-2115918.5	-2115914.4	-2115916.7	-2115920.4
**5**	-2115916.5	-2115917.0	-2115923.6	-2115918.3	-2115912.5

Table S6 Estimates for demographic parameters based on coalescent simulations. Estimates are shown for the ten best-fitting sets of simulations out of 80 sets of simulations with the software FASTSIMCOAL2. (A) Estimates including all Atlantic (USA, CAP, IVO) and the Indo-Pacific individuals (IND, JAP, ELS), (B) estimates without the heterogenic ELS sample. Note that migration rates are given forward in time, per generation, and that the divergence time (div. time) is also given in units of generations. N_e, effective population size; st.dev., standard deviation.

A) Atlantic (USA, CAP, IVO) vs Indo-Pacific (IND, JAP, ELS)

	N_e				migration	
**Replicate**	**ancestral**	**Atlantic**	**IndoPac**	**div. time**	**Atlantic-IndoPac**	**IndoPac-Atlantic**
1	23289	3128	34548	24603	0.004	0.033
2	21522	2406	36315	31473	0.001	0.034
3	856	1075	10799	19001	0.010	0.076
4	24426	1476	45319	39787	0.000	0.055
5	39456	1790	61704	30305	0.004	0.049
6	30728	2126	53795	42078	0.004	0.037
7	46140	1284	69964	39546	0.000	0.063
8	47284	1937	70009	38385	0.002	0.053
9	43443	2580	63146	30924	0.004	0.034
10	54076	1750	78633	39541	0.000	0.045
**mean**	**33122.0**	**1955.2**	**52423.2**	**33564.3**	**0.003**	**0.048**
**st.dev.**	**16018.9**	**625.6**	**20733.6**	**7603.4**	**0.003**	**0.014**

B) without ELS

	N_e				migration	
**Replicate**	**ancestral**	**Atlantic**	**IndoPac**	**div. time**	**Atlantic-IndoPac**	**IndoPac-Atlantic**
1	21474	1257	39371	33910	0.006	0.071
2	9259	1587	19580	21517	0.008	0.049
3	41636	1388	64488	38971	0.003	0.060
4	55190	2686	90883	51289	0.004	0.034
5	24580	2166	38733	24683	0.004	0.042
6	37989	3844	56278	42896	0.003	0.026
7	34568	3991	53327	41231	0.003	0.021
8	32804	2773	53582	38076	0.004	0.039
9	41285	4512	59457	40864	0.002	0.018
10	52375	4283	72153	38341	0.002	0.016
**mean**	**35116.0**	**2848.7**	**54785.2**	**37177.8**	**0.004**	**0.038**
**st.dev.**	**13988.1**	**1241.7**	**19610.5**	**8687.6**	**0.002**	**0.018**
